# Supplementary material for: Clinical Comparison Between Curative and Non-Curative Treatment for Hepatocellular Carcinoma with Hepatic Vein Invasion: A Nationwide Cohort Study
Source: Cancers (Basel). 2025 May 27;17(11):1794. doi: 10.3390/cancers17111794 (PMC12153590; doi:10.3390/cancers17111794)
Supplement: Supplementary file 1 [file cancers-17-01794-s001.zip › cancers-3638351-supplementary.pdf]

Standardized Mean Differences (Treated - Control)						
**Variable**	**Observations**	**Mean Difference**	**Standard Deviation**	**Standardized Difference**	**Percent Reduction**	**Variance Ratio**
**Logit Prop score**	All	1.00607	1.03618	0.97094		0.5508
	Region	0.97954		0.94534	2.64	0.5743
	Matched	0.22403		0.21621	77.73	1.1186
**Tumor size**	All	-1.9109	4.432479	-0.43111		1.1468
	Region	-1.83312		-0.41356	4.07	1.1739
	Matched	-1.19394		-0.26936	37.52	1.0981
**Tumor number**	All	-1.16024	1.463722	-0.79266		0.3772
	Region	-1.13564		-0.77586	2.12	0.3808
	Matched	-0.08067		-0.05512	93.05	1.1323
**ALBI grade**	All	-0.20452	0.446168	-0.4584		0.6577
	Region	-0.19841		-0.4447	2.99	0.6613
	Matched	-0.07166		-0.16061	64.96	0.8087

Standard deviation of All observations used to compute standardized differences

Table S1: Means, standard deviations, and standardized mean differences on covariates and propensity scores post-matching. Abbreviations: ALBI, albumin-bilirubin

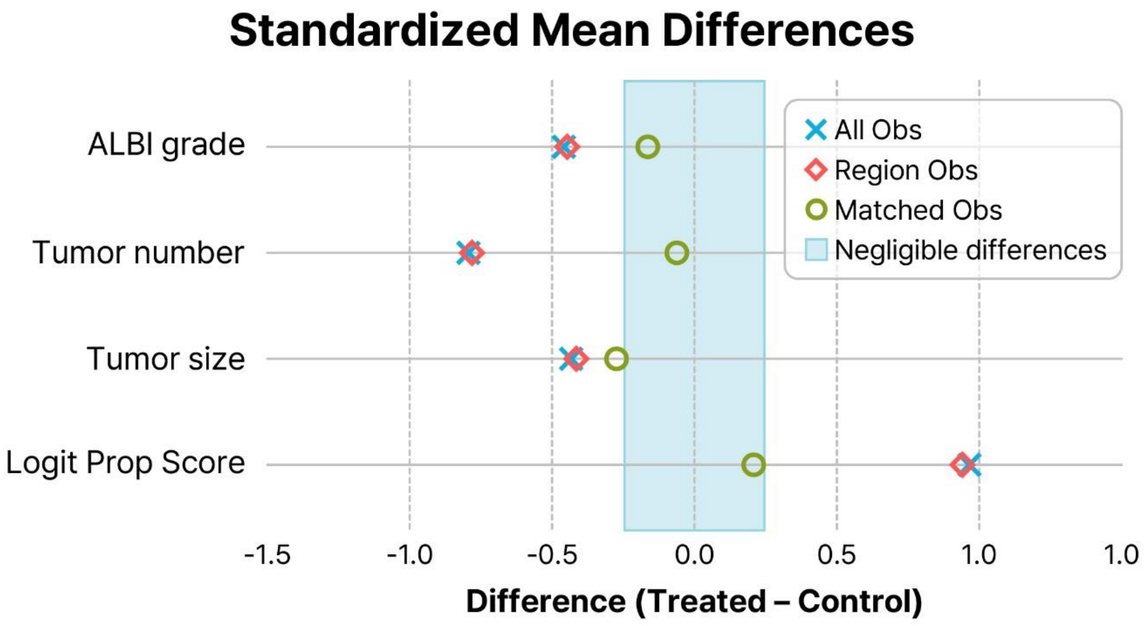

Figure S1: Standardized mean differences before and after matching**.** Abbreviations: ALBI, albumin-bilirubin

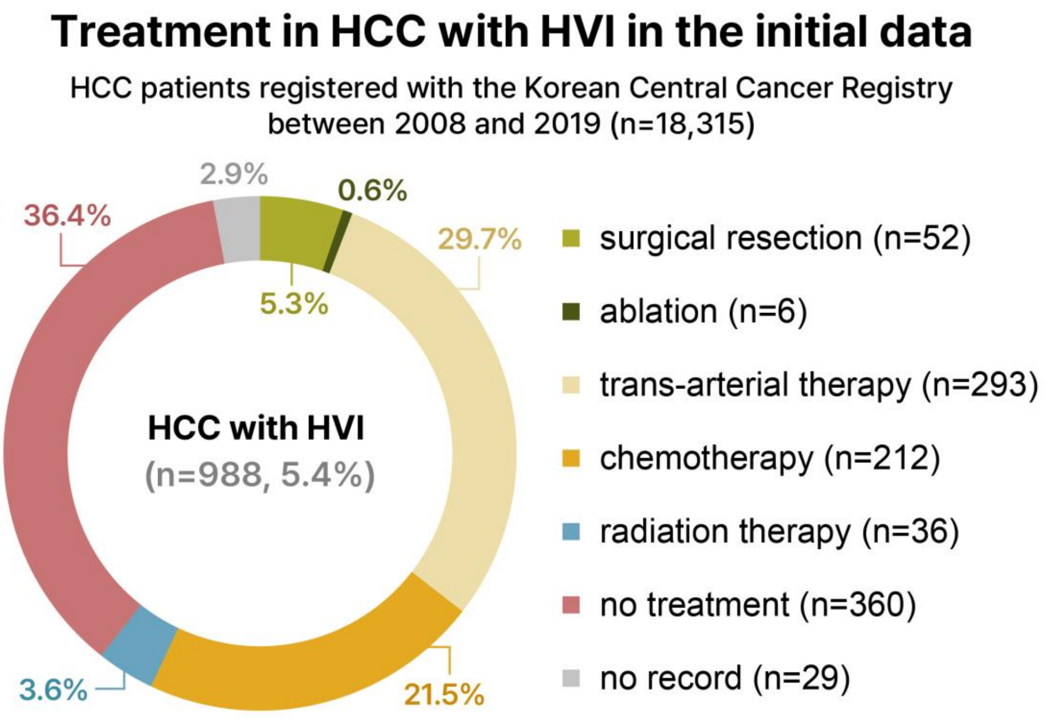

Figure S2: Distribution of treatment methods in hepatocellular carcinoma with hepatic vein invasion in the entire cohort before applying exclusion criteria and propensity score matching. Abbreviations: HCC, hepatocellular carcinoma; HVI, hepatic vein invasion

**33 patients of HCC with HVI who did not receive any treatment despite having Child-Pugh class A, ECOG performance status 0-1, and no distant metastasis**			
Variable	Median (IQR) or N (%)	Variable	Median (IQR) or N (%)
Age	63 (56 – 72)	MELD score	8 (7 – 9)
Sex (female)	6 (19.2%)	PT-INR	1.06 (1.00 – 1.14)
BMI (kg/m^2)	21.97 (20.77 – 25.40)	Cr (mg/dL)	0.80 (0.71 – 1.00)
Ascites	5 (15.1%)	Tumor number	1 (1 – 5)
Hepatic encephalopathy	0 (0%)	Tumor size (cm, max)	12.0 (8.5 – 15.4)
Albumin (g/dL)	3.8 (3.6 – 4.1)	Portal vein invasion	26 (78.7%)
Total bilirubin (mg/dL)	0.88 (0.60 – 1.30)	Hepatic artery invasion	0 (0%)
ALBI score	-2.56 (-2.73 – -2.19)	AFP (ng/mL)	201.5 (10.4 - 10500.0)
ALBI grade (2)	19 (57.5%)	PIVKA-II (mAU/mL)	11506.5 (1400.0 - 37212.4)

Table S2. The baseline characteristics of the 33 patients of HCC with HVI who did not receive any treatment despite having Child-Pugh class A, PST 0-1, and no distant metastasis. Values are presented as median (IQR) for continuous data and n (%) for categorical data. Abbreviations: ECOG, Eastern Cooperative Oncology Group; BMI, body mass index; ALBI, albumin-bilirubin; MELD, model for end-stage liver disease; PT-INR, prothrombin time-international normalized ratio; Cr, creatinine; AFP, alpha-fetoprotein; PIVKA-II, protein induced by vitamin K absence or antagonist-II.

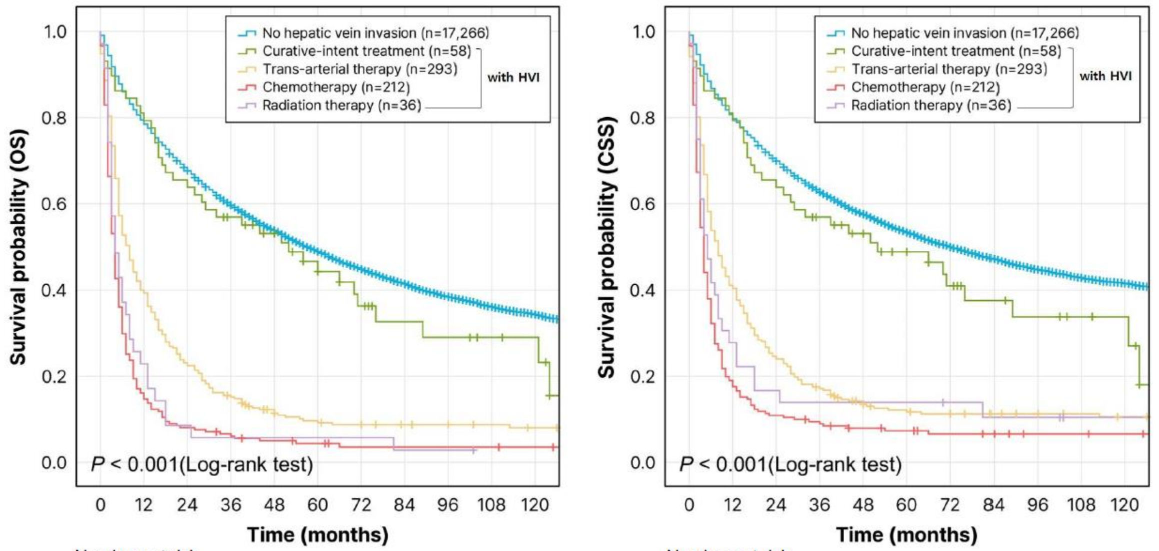

Numbers at risk

17,266	9,536	6,750	4,479	2,903	1,782
58	37	25	13	8	5
293	65	26	18	13	10
212	17	9	4	4	3
36	3	3	3	1	0

(a)

Numbers at risk

17,266	9,853	7,225	4,991	3,351	2,114
58	37	25	15	9	5
293	70	32	24	17	14
212	23	14	9	5	4
36	6	5	4	3	1

(b)

Figure S3: Overall and cancer-specific survival according to treatment methods in the entire cohort before applying exclusion criteria and propensity score matching. (**a**) OS; (**b**) CSS. Abbreviations: OS, overall survival; CSS, cancer-specific survival. A survival curve of hepatocellular carcinoma with no hepatic vein invasion is presented regardless of treatment modality. The survival curves of hepatocellular carcinoma with hepatic vein invasion are shown in different colors according to treatment modality**.** Abbreviations: OS, overall survival; CSS, cancer-specific survival; HVI, hepatic vein invasion
